# Extraction of guide wire breakage from pedicle channel during percutaneous endoscopic posterior lumbar interbody fusion: a case report

**DOI:** 10.1093/jscr/rjaf278

**Published:** 2025-05-07

**Authors:** Cheng Li, Yangyang Guo

**Affiliations:** Department of Spine Surgery, Wangjing Hospital, China Academy of Chinese Medical Sciences, National Center for Traditional Chinese Medicine in Orthopedics and Traumatology, No. 6, South Zhonghuan Road, Chaoyang District, Beijing, 100102, China; Department of Spine Surgery, Zhoukou Orthopedic Hospital, Taihao Road, Zhoukou City, Henan Province, 466000, China; Department of Spine Surgery, Zhoukou Orthopedic Hospital, Taihao Road, Zhoukou City, Henan Province, 466000, China

**Keywords:** guidewire breakage, lumbar interbody fusion, case report, intraoperative complication, endoscopic surgery

## Abstract

This report presents a rare case of guidewire fracture during percutaneous endoscopic posterior lumbar interbody fusion (PE-PLIF) and describes the successful retrieval technique. A 49-year-old male with lumbar stenosis and spondylolisthesis underwent PE-PLIF, during which fluoroscopy identified a fractured guidewire within the vertebral body. The fragment was retrieved using endoscopic grasping forceps after removing the pedicle screw, and complete removal was confirmed by fluoroscopy. This case highlights the importance of thorough instrument inspection and careful intraoperative handling to prevent and manage such rare complications in spinal surgery.

## Introduction

In spinal surgery, instrument breakage represents a rare but serious complication [1–4]. With the continuous development of minimally invasive spinal techniques and the evolution of surgical concepts, instruments have become increasingly refined to meet the demands of complex surgical procedures. However, this refinement has also made them more prone to breakage. In the confined space of endoscopic surgery, retrieving broken instruments poses a considerable challenge. This case report describes the fracture of a guide wire during a percutaneous endoscopic posterior lumbar interbody fusion (PE-PLIF) procedure and details its successful identification and extraction from the vertebral body.

## Case report

**Figure 1:**
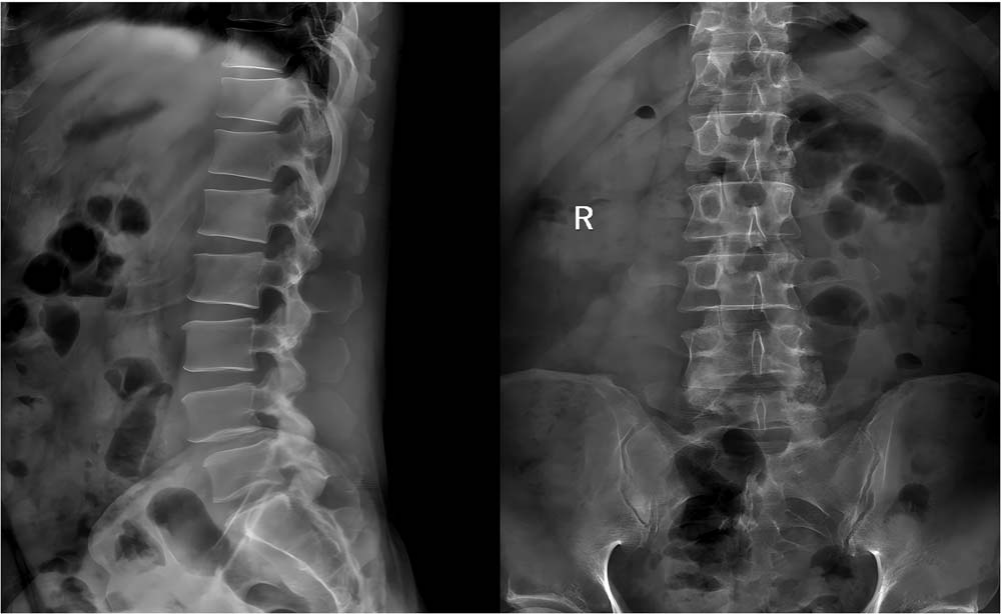
Preoperative anteroposterior and lateral radiographs of the lumbar spine.

A 49-year-old male presented with a 10-month history of low back pain accompanied by numbness in the left lower limb. Over the past 2 weeks, his symptoms had progressively worsened without any identifiable precipitating factor, with increasing pain and numbness radiating down the left lower extremity. The patient reported that walking about 20 meters aggravated his symptoms, which were relieved after rest, allowing him to walk another short distance before the pain recurred. Due to the lack of significant relief from over-the-counter analgesics and topical medications, he presented to our outpatient clinic for further evaluation. Clinical examination and preoperative imaging demonstrated lumbar spinal stenosis at the L4/L5 level, lumbar instability, and grade I spondylolisthesis at L4 (Fig. 1). Following a comprehensive preoperative assessment, the patient was scheduled for PE-PLIF.

**Figure 2:**
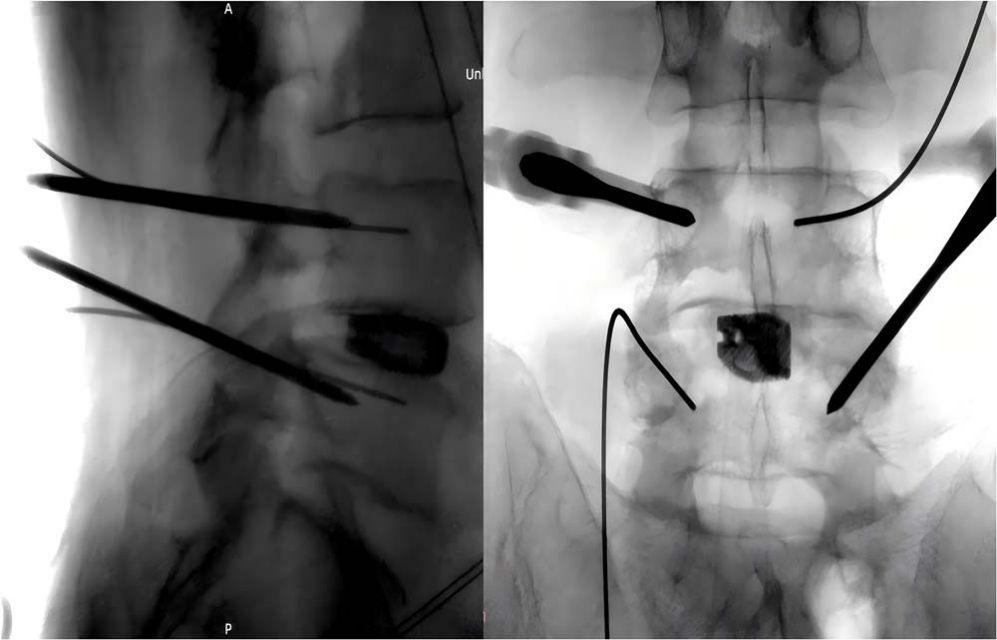
Intraoperative placement of the pedicle screw.

**Figure 3:**
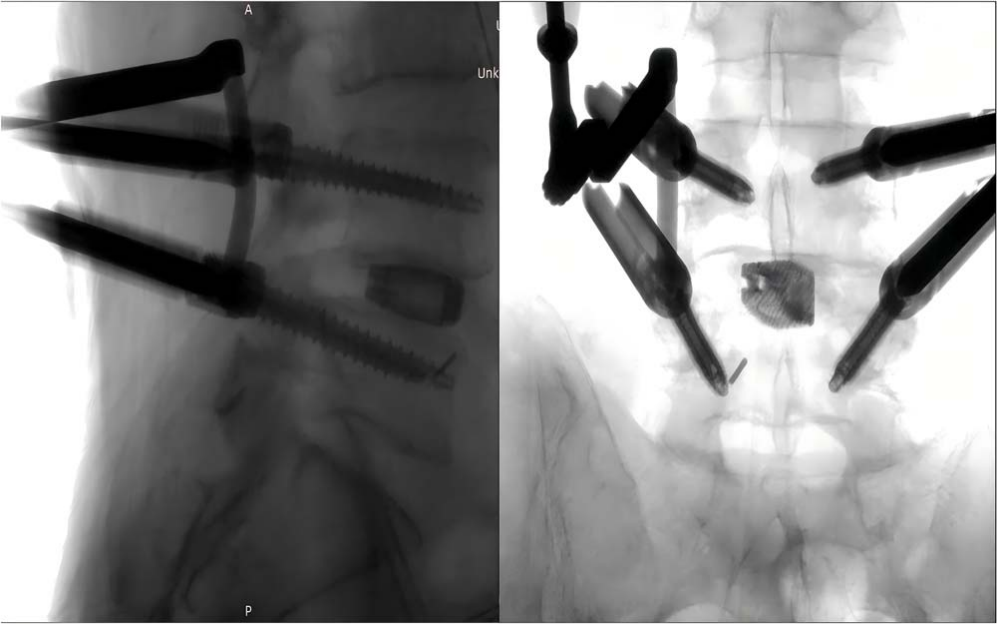
Intraoperative fluoroscopic confirmation of guidewire breakage.

During the surgery, after sufficient decompression at the L4/L5 level and placement of the interbody fusion cage, a complication occurred during the percutaneous pedicle screw insertion. Intraoperative G-arm fluoroscopy confirmed the correct trajectory of the pedicle screw, established by the guidewire and puncture needle, and the pedicle screw was inserted along the established pathway (Fig. 2). However, after removing the guidewire, fluoroscopic imaging revealed that a portion of the guidewire had broken near the anterior margin of the left L5 pedicle screw (Fig. 3). The fracture likely occurred during screw insertion or tightening, which subsequently displaced the broken guidewire toward the anterior vertebral margin. Given the proximity of the fractured guide wire to the anterior vertebral margin, along with the risks of future migration causing injury to adjacent tissues or vessels, as well as the potential for local infection or pain, the decision was made to remove the fractured guidewire after obtaining informed consent from the patient’s legal representative.

**Figure 4:**
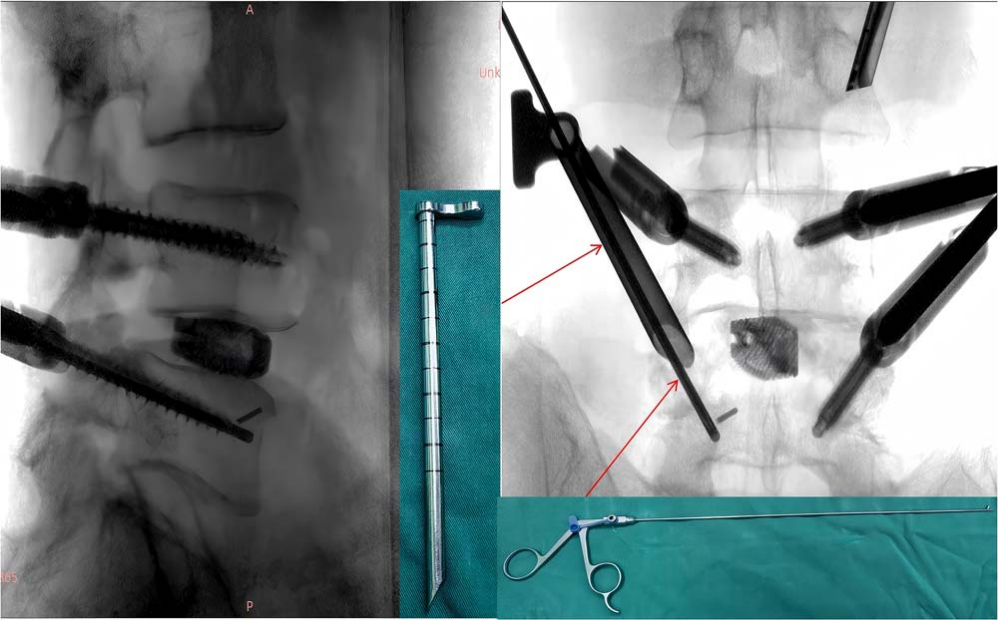
Endoscopic forceps localizing the fractured guidewire.

**Figure 5:**
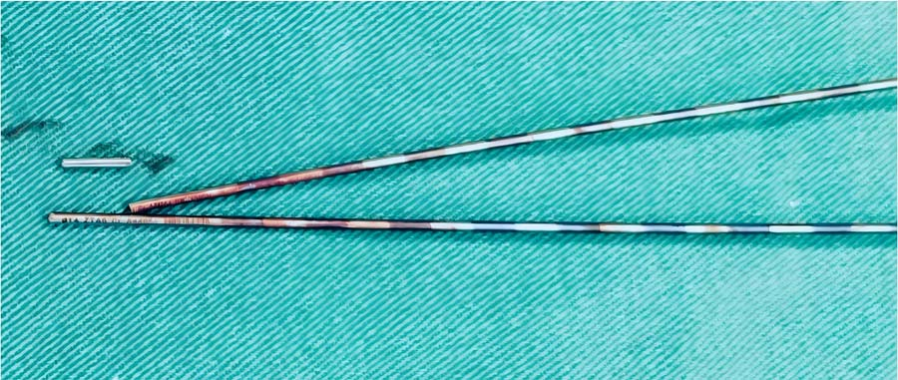
The fractured guidewire during surgery.

Following removal of the left L5 pedicle screw during the procedure, the smallest endoscopic sheath was inserted along with the endoscope to explore the screw trajectory. Under fluoroscopic guidance, endoscopic forcep was utilized to identify and grasp the fractured guidewire (Fig. 4). Tactile feedback of a metallic sensation confirmed precise localization. Approximately 6 mm of the fractured guidewire was successfully extracted (Fig. 5). Subsequent fluoroscopy confirmed complete removal of the broken guidewire. The left L5 pedicle screw was reinserted, and bilateral connecting rods were placed. The nuts were then securely fastened. Hemostasis was confirmed under endoscopic visualization, and the surgical incisions were closed and dressed with sterile coverings.

**Figure 6:**
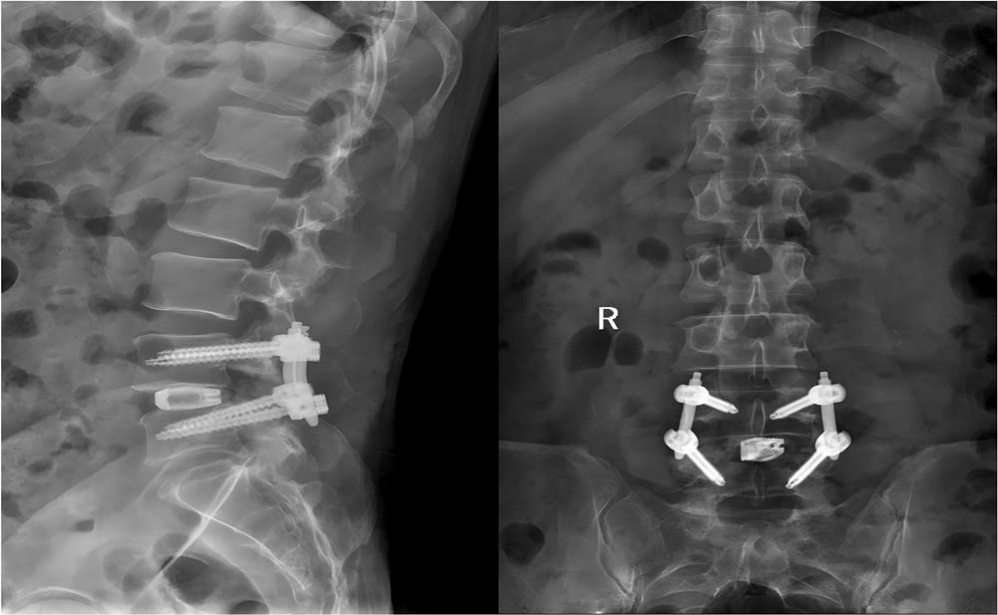
Postoperative anteroposterior and lateral radiographs of the lumbar spine.

Following the surgery, the patient remained in stable condition with no reported discomfort. Postoperative radiographs verified complete extraction of the guidewire, with no residual fragments observed at the fracture site (Fig. 6). The patient experienced an uneventful recovery, and subsequent follow-up evaluations demonstrated satisfactory outcomes.

## Discussion

In orthopedic surgery, the incidence of instrument breakage ranges from 0.21% to 0.45% [5–7]. In a study on instrument breakage in orthopedic procedures, Pichler *et al.* [7] reported 42 instances of instrument failure, of which only five were successfully removed. During follow-up, none of the patients exhibited any symptoms; however, over half of the cases involved traumatic injuries. Compared to other orthopedic sites, the presence of residual metal fragments in spinal surgery poses heightened risks due to the spine's unique anatomy. Instrument breakage during posterior approaches can result in significant neurological complications [3], while anterior spinal procedures may endanger vascular structures and visceral organs [8, 9]. Guan *et al.* [10] reported two successful cases of guide wire retrieval during percutaneous endoscopic lumbar discectomy. However, these reports did not address the involvement of deep vertebral structures. Čeleš *et al.* [4] described a case where a surgical blade fractured within the intervertebral space during L5-S1 discectomy and transforaminal interbody cage implantation. After multiple failed retrieval attempts through the posterior approach, an anterior laparoscopic approach was ultimately employed in a subsequent surgery to extract the broken blade. Zhao *et al.* [1] reported three cases of guide wire breakage in their study on percutaneous pedicle screw fixation for treating thoracolumbar and lumbar fractures. In two cases, the guide wire was retrieved by removing the screws, while in one case; the guide wire could not be removed and remained permanently within the vertebral body. However, the authors did not provide detailed descriptions of the specific retrieval techniques employed. This study presents a method for retrieving a broken guide wire within the vertebral body during PE-PLIF. Upon recognizing the breakage intraoperatively, the location of the fractured guide wire was confirmed using G-arm fluoroscopy. Endoscopic forceps were used to grasp the fractured guidewire in alignment based on its orientation, with careful intraoperative assessment for tactile metallic feedback.

The anatomy of the spine is complex, with major neurovascular structures in close proximity to the operative field. Therefore, any instrument breakage during spinal procedures necessitates prompt retrieval to prevent potential complications. The presence of a broken guidewire can lead to serious issues, including nerve damage, vascular injury, and increased risk of infection. Unfortunately, there remains a lack of specialized tools designed specifically for the extraction of broken fragments in spinal procedures.

Comprehensive preoperative and postoperative checks are critical. Surgeons must thoroughly assess the condition of reusable instruments, particularly those not designated as single-use, to minimize the risk of breakage during procedures. Regular maintenance and inspection protocols are essential to detect early signs of wear or fatigue, preventing instrument failure. This proactive strategy helps reduce the likelihood of complications. In addition to instrument integrity, surgical technique plays a crucial role in preventing instrument breakage, including methods to minimize excessive manipulation and avoid applying undue force during insertion or advancement.

The extraction of a broken instrument during spinal surgery presents significant challenges but underscores the importance of advancing surgical practices, instrument design, and specialized training. Enhancing surgical training, refining instrument design, and developing specialized retrieval techniques are essential for reducing complications related to instrument failure and optimizing patient outcomes in spinal surgery.
